# Supplementary material for: Could a ring treatment approach be proposed to control Taenia solium transmission in a post elimination setting? A pilot study in Zambia
Source: PLoS Negl Trop Dis. 2024 Aug 7;18(8):e0012400. doi: 10.1371/journal.pntd.0012400 (PMC11346962; doi:10.1371/journal.pntd.0012400)
Supplement: Attachment — Submitted filename: Peer review_Trevisan et al.pdf [file pntd.0012400.s001.pdf]

Could a ring treatment approach be proposed to control *Taenia solium* transmission in a post elimination setting? A pilot study in Zambia

(Trevisan et al)

In this interesting research paper authors presented if they could implement a new *Taenia solium (TS)* -spread control strategy (used in Peru) in a **post-eliminated** setting in Zambia. Method consists of – 1) identifying the TS carrier by testing blood from pigs and stool from humans; 2) forming a ring of 50 m radius encircling the seropositive pig or copro-ELISA positive human; 3) treating the pigs and humans living within each ring. Outcomes were considered the willingness of people **to give sample and to get treated**.

This research has addressed many important issues regarding the implementation of new strategy in a community who already were the part of another TS-control program. **The main strength of this article, I think, is that the authors claimed it is such first approach in sub-Saharan Africa and authors clearly described the limitation of this research**. However, respecting the efforts and hardship of doing such study, I think, it will be better to reconsider the publication of this article in PLOS-NTD as the concerns are -

.

Major points:

1. The outcome measure used in this study is ONLY (one) behavior ("willingness") of study participants. The willingness of people of a ***sensitized community*** is expected to be very subjective. It can be influenced by the information they were provided with (such as – the presence of infected pigs or humans near to them), living within the ring, knowledge about the success of the previously implemented strategy. Of note, the authors already have stated that concern in discussion.
2. Generally speaking and the authors also discussed, if we apply a new control strategy (for TS control) within a study population or a study area where already one control strategy was successfully (as per authors' claim) implemented, it is expected that the people will response positively to the new strategy, mainly because they are already sensitized and also they were informed about the health threat from TS carrier. This lead to the overestimation of the willingness of people to accept new strategy (or even other notions can evolve such as – people of that community may think that "the previous strategy did not work well so it is better to participate in new strategy"). Then, also questions arise –
    a. ***What is the point of adopting a new strategy in an area where "elimination of active T. solium transmission was achieved" ?*** Even if it is justified by the notion that to follow up the sustainability of the success authors achieved from previous strategy, still ***why are they preferring new strategy over existing strategy to implement on that study area?***
    b. ***Why did the authors find so many pigs infected with parasites if the spread of the parasite "has been successfully stopped" previously?*** (I am posing this question, as authors said they did an Ag-ELISA in pig blood which identifies pigs with viable cysticerci). Okey, authors said that "antibodies remain positive long after treatment". ***How long? Can they give that information with reference?*** In any case, it will be better to state what was the time gap between treatment (MDA for previous control strategy) and serological test, otherwise "why you got so many

(6/47) pigs were seropositive during follow-up period 4" will not be justified. I am not sure what are the sensitivity and specificity of the tests authors used.

3. I see the authors mentioned "**elimination** of active *T. solium* transmission was achieved" (page 2), "the spread of the parasite had been successfully **stopped** (page 3)", "active *T. solium* transmission in pigs was **interrupted** (page 5)". That's confusing. ***Which argument should be correct one?*** given that first two arguments mean complete (100%) cessation of transmission in the study area while last one indicates, the transmission process was interrupted but process is still existing there.
4. If I am not wrong, the follow up sampling was the part of monitoring (of previous control program), not solely for implementing new strategy, even if I am wrong, authors distributed pots to the people who already has agreed ("willing") to take part to the project, so, ***How people's "willingness to be samples" can be considered as an outcome measure?*** I think it is just the proportion of sample recovery/receiving.

Minor points:

1. ***Page-2/ Section "Methodology/Principal findings:"/Line 26:*** I think, it will be good idea to make separate sections, one for "Methodology" and another for "Principal findings". Or use the section sub-heading as "Methodology and principal findings". I guess, here, "/" symbol referring "or" which is misleading.
2. ***Page-14/ "Discussion"/L277-279:*** The conclusion drawn by the authors is a concern. It is difficult to say "a 50-meter radius" would be best option based on only one study in one type of setting.

Other editing:

1. ***Page-3/Section "Authors summary"/Line 49-50:*** People usually refer the *Taenia solium* as "the pork tapeworm" even though, generally, we don't find "adult tapeworm" in pork or pig rather in human with taeniasis. Can we think in any different way?
2. ***Page-4/Section "Introduction" /L67-69:*** First sentence needs revision. Are the authors referring "control or elimination" strategies or tools? Again, Control of TS in what/where? Or elimination of TS from what/where? It is necessary to blend the idea with second sentence "Highly efficient control tools………".
3. ***Page-17/Section "References" /L401-402:*** Where did the authors used this reference within the text?
