# Supplementary material for: Could a ring treatment approach be proposed to control Taenia solium transmission in a post elimination setting? A pilot study in Zambia
Source: PLoS Negl Trop Dis. 2024 Aug 7;18(8):e0012400. doi: 10.1371/journal.pntd.0012400 (PMC11346962; doi:10.1371/journal.pntd.0012400)
Supplement: Attachment — Submitted filename: R1.1_Response to Reviewers comments_03072024.pdf [file pntd.0012400.s002.pdf]

INSTITUTE OF TROPICAL MEDICINE ANTWERP

Dear reviewers,

Thank you for your thorough reading and constructive comments and suggestions. Please find a response in this document and an adapted and clean version of the manuscript. We hope we have addressed all the comments sufficiently and you deem our manuscript ready for publication.

Reviewer #1:

The manuscript PNTD-D-24-00616 is well written and clear in its statements. I do not understand why the ring elimination method was used to evaluate areas in Zambia that have been already controlled for *Taenia solium* cysticercosis and taeniasis, the authors have to explain the reasoning since the approaches used are expensive and time consuming:

Dear reviewer, thank you for your positive feedback and comment. Based on transmission model simulations, we could estimate that with a 2-year integrated One Health approach eliminating *T. solium* transmission would be possible. This was demonstrated in our paper "Gabriël S, Mwape KE, Hobbs EC, Devleesschauwer B, Van Damme I, Zulu G, et al. Potential elimination of active *Taenia solium* transmission in Africa. N Engl J Med. 2020;383: 396–397. doi:10.1056/NEJMc1909955", however based on our model predictions, once the programme would end, the prevalence of human taeniosis and porcine cysticercosis would start to rise again. This was the rationale for this study, where we wanted to explore if a ring treatment strategy (similar to the one trialled in Peru) could be proposed in a sub-Saharan setting, to maintain control of parasite transmission. Before future recommendations on such an approach can be suggested to health authorities, it was necessary to assess the approach and the overall willingness of the local community residents and farmers to participate in such an approach, despite its cost and time constraints, which would be estimated in a later stage of the project.

Reviewer #2:

Dear reviewer, thank you for the thorough feedback and suggestions provided. We hope

our answers will be satisfactory and that you deem our manuscript ready for publication.

Major Points

1) The outcome measure used in this study is ONLY (one) behavior ("willingness") of study participants. The willingness of people of a sensitized community is expected to be very subjective. It can be influenced by the information they were provided with (such as – the presence of infected pigs or humans near to them), living within the ring, knowledge about the success of the previously implemented strategy. Of note, the authors already have stated that concern in discussion.

Thank you for your comment. Indeed, this study also emphasises the importance of a good prior sensitization and trust relation with the affected populations. We also clearly describe that we study the implementation of a ring strategy in a post elimination/control setting, whereby the community was already sensitised. Indeed, if people know that there has been/is an infected pig/person nearby, this may impact their willingness, and this is exactly an important part of the ring strategy, that people within the ring are informed on a positive case, to enhance compliance.

2) Generally speaking and the authors also discussed, if we apply a new control strategy (for TS control) within a study population or a study area where already one control strategy was successfully (as per authors' claim) implemented, it is expected that the people will response positively to the new strategy, mainly because they are already sensitized and also they were informed about the health threat from TS carrier. This lead to the overestimation of the willingness of people to accept new strategy (or even other notions can evolve such as – people of that community may think that "the previous strategy did not work well so it is better to participate in new strategy"). Then, also questions arise –.

The first strategy implemented was meant to eliminate parasite transmission, while the strategy applied in this study would serve to control parasite transmission and avoid a future rise of the prevalence as shown by the simulations of our transmission model. "Gabriël S., Mwape K.E., Phiri I.K., Devleesschauwer B. and Dorny P. *Taenia solium* control in Zambia: The potholed road to success. Par. Epidemiol Cont. 2019 Feb; 4: e00082. doi:10.1016/j.parepi.2018.e00082". Willingness and feasibility are assessed in this post elimination setting.

a) What is the point of adopting a new strategy in an area where "elimination of active T. solium transmission was achieved"? Even if it is justified by the notion that to follow up the sustainability of the success authors achieved from previous strategy, still why are they preferring new strategy over existing strategy to implement on that study area?

With the 2-year integrated One Health approach we demonstrated that eliminating *T. solium* transmission would be possible, however as mentioned to reviewer one, the same model used to design the interventions, also showed that if nothing else would be done in an area, the prevalence would rise again. Besides the fact that such an intensive approach would not be sustainable in the long term. Therefore, we wanted to explore if a ring treatment strategy (similar to the one trialled in Peru) could be proposed in a sub-Saharan setting. To continue the same strategy by which active elimination of transmission in pigs was achieved, which included an intensive combination of 4 monthly interventions targeting the human and pig host, would not be feasible.

b) Why did the authors find so many pigs infected with parasites if the spread of the parasite "has been successfully stopped" previously? (I am posing this question, as authors said they did an Ag-ELISA in pig blood which identifies pigs with viable cysticerci). Okey, authors said that "antibodies remain positive long after treatment". How long? Can they give that information with reference? In any case, it will be better to state what was the time gap between treatment (MDA for previous control strategy) and serological test, otherwise "why you got so many during follow-up period 4" will not be justified. I am not sure what are the sensitivity and specificity of the tests authors used.

Thanks for the valid observation. As simulated in the model, and shown in our study, two-year intensive interventions could eliminate active transmission (no pig with viable cysticerci was identified in the post intervention survey conducted in December 2017). However, human MDA coverage was not 100%, therefore possible tapeworm carriers were still living in the study area. As open defecation is a common practice, it is possible that some pigs got re-infected soon after. As simulated and recommended by the model, the follow up samplings started six months later, with half year intervals to monitor the

potential rise of prevalence. The ring strategy was designed, to control parasite transmission in future. We added serology (B158/B60 serum Ag ELISA (se 0.867-sp 0.947) because of the higher sensitivity compared to tongue palpation (se 0.210-sp 1.000) (Dorny et al 2004). However, this might have led to false positive pigs as the test has shown to cross-react with *Taenia hydatigena*, another porcine parasite present in the area (Chembensofu et al 2017). Furthermore, we observed intensive movement of pigs and people entering the study area from outside, representing ample opportunities for bringing infection (either porcine cysticercosis or human tapeworm carriers) into the communities. Two and a half years passed between the last MDA and follow-up period 4.

3) I see the authors mentioned "elimination of active T. solium transmission was achieved" (page 2), "the spread of the parasite had been successfully stopped (page 3)", "active T. solium transmission in pigs was interrupted (page 5)". That's confusing. Which argument should be correct one? given that first two arguments mean complete (100%) cessation of transmission in the study area while last one indicates, the transmission process was interrupted but process is still existing there.

Thank you for this observation. We could demonstrate elimination of active *T. solium* transmission as no more pig with viable cysticerci was found in the area by the time of the post intervention survey in December 2017. The spread of the parasite via infected meat was stopped, hence active transmission was eliminated. This was changed also in the sentence on page 5. The sentence now reads as follows: "This study was conducted in the Nyembe neighbourhood, in the Katete District, the study arm where active *T. solium* transmission in pigs was eliminated."

4) If I am not wrong, the follow up sampling was the part of monitoring (of previous control program), not solely for implementing new strategy, even if I am wrong, authors distributed pots to the people who already has agreed ("willing") to take part to the project, so, How people's "willingness to be samples" can be considered as an outcome measure? I think it is just the proportion of sample recovery/receiving..

This study was indeed included as part of the monitoring phase after elimination of active *T. solium* transmission was achieved. The participants had each time to agree to participate to the sampling before the pots were distributed and samples taken. The

study participants also had to agree and be willing to get treatment after being informed on the ring strategy.

Minor points:

1. Page-2/ Section "Methodology/Principal findings:"/Line 26: I think, it will be good idea to make separate sections, one, for "Methodology" and another for "Principal findings". Or use the section sub-heading as "Methodology and principal findings". I guess, here, "/" symbol referring "or" which is misleading.

Thank you for the suggestion. The word 'and' was added. Now the heading reads as follows: "Methodology and Principal findings".

2. Page-14/ "Discussion"/L277-279: The conclusion drawn by the authors is a concern. It is difficult to say "a 50-meter radius" would be best option based on only one study in one type of setting.

Thank you for the observation. Indeed, the radius should be based on the area characteristics and demographics. The word '50-meter' was replaced by the word 'certain' radius. Now the sentence reads as follows: "This would imply that whenever a pig is found or reported infected, the household members and all pigs living within a certain radius would be treated".

Other editing:

1. Page-3/Section "Authors summary"/Line 49-50: People usually refer the Taenia solium as "the pork tapeworm" even though, generally, we don't find "adult tapeworm" in pork or pig rather in human with taeniasis. Can we think in any different way?

Thank you for your remark. We now tried to add the word parasite to pork tapeworm in the first sentence of the Author summary and use tapeworm larvae instead of parasite in the second sentence. The paragraph now reads as follows: *"In this study, we looked at whether a method used to control the spread of the pork tapeworm, a parasite transmitted between pigs and people, in Peru could also work in Zambia. The method involved treating people and pigs living near animals infected with tapeworm larvae."*

We find it important to name the tapeworm according to the "origin" as there are multiple tapeworms that can infect humans, like for example the beef tapeworm, fox tapeworm, fish tapeworm etc.

2. Page-4/Section "Introduction" /L67-69: First sentence needs revision. Are the authors referring "control or elimination" strategies or tools? Again, Control of TS in what/where? Or elimination of TS from what/where? It is necessary to blend the idea with second sentence "Highly efficient control tools………".

Thank you for your suggestions. The sentence was indeed confusing and is modified to give more clarity. Now it reads as follows: *"Over the last decade, highly efficient Taenia solium intervention tools have been developed and evidence-based control/elimination strategies have been trialled".*

3. Page-17/Section "References" /L401-402: Where did the authors used this reference within the text?

Thank you for your observation. Indeed, reference 20 is the same one as reference 2 and does not appear in the text. This duplicate reference was removed and reference numbers 21 and 22 adapted.

We hope we addressed your comments adequately and look very much look forward hearing from you.

Yours sincerely,
Chiara Trevisan

Chiara Trevisan, PhD
Researcher
Institute of Tropical Medicine (ITM)
Department of Public Health
Nationalestraat 155
2000 Antwerp, BELGIUM
E-mail: ctrevisan@itg.be
